# Social Protection and Social Cohesion in Times of the COVID-19 Pandemic: Evidence from Kenya

**DOI:** 10.1057/s41287-022-00541-1

**Published:** 2022-05-12

**Authors:** Christoph Strupat

**Affiliations:** German Development Institute/Deutsches Institut für Entwicklungspolitik, Tulpenfeld 6, 53111 Bonn, Germany

**Keywords:** Social protection, Social assistance, Social cohesion, COVID-19, Kenya, I38, O17

## Abstract

This paper examines empirically whether social protection in the form of social assistance programmes are affecting social cohesion during the COVID-19 pandemic. Using unique primary data from nationally representative, in-person surveys from Kenya allows for the exploration of the effect of social protection on attributes of social cohesion. The analysis employs a difference-in-differences approach that compares households with and without social assistance coverage before and after the first wave of the pandemic. The main findings show that social assistance does not influence attributes of social cohesion. One potential explanation of this result is that social assistance benefits were in general too small to entirely offset the negative economic consequences of the pandemic. Overall, these results point to the limitations of social assistance programmes that do not necessarily affect social cohesion in times of large covariate shocks, such as the COVID-19 pandemic.

## Résumé

Cet article cherche à savoir de façon empirique si la protection sociale - sous forme de programmes d'aide sociale - affecte la cohésion sociale dans le cadre de la pandémie de COVID-19. L'utilisation de données primaires uniques provenant d'enquêtes en personne, représentatives au niveau national, permet d'explorer l'effet de la protection sociale sur les attributs de la cohésion sociale au Kenya. L'analyse utilise la méthode des doubles différences qui compare les ménages avec et sans aide sociale avant et après la première vague de la pandémie. Les principaux résultats montrent que l'aide sociale n'influence pas les attributs de la cohésion sociale. Il est possible d’expliquer ce résultat par le fait que les aides sociales étaient en général trop faibles pour compenser entièrement les conséquences économiques négatives de la pandémie. Dans l'ensemble, ces résultats soulignent les limites des programmes d'aide sociale qui n'affectent pas nécessairement la cohésion sociale en période d’importants chocs covariants, comme la pandémie de COVID-19.

## Introduction

The COVID‐19 pandemic is a major public health challenge that is generating serious economic and social impacts that are likely to persist for some time. In order to mitigate the adverse economic consequences of the pandemic and the related containment policies, social protection programmes have been adapted and expanded on a large scale in many countries (Gentilini et al. 2021). Initial studies have shown that these measures have been effective in reducing some of the negative economic impacts of the pandemic, including poverty, hunger and inequality (Abay et al. 2021; Banerjee et al. 2020; Bottan et al. 2021; Lustig et al. 2020). However, in the past years, the goals of social protection have been expanded, and it has been recognised that social protection can also affect more complex outcomes, such as human capital, health and social cohesion (Garcia-Mandicó et al. 2021; Koehler 2021; Strupat 2021). The literature on the relationship with social cohesion is still limited and does not consider covariate shocks, such as the COVID-19 pandemic, that alone can affect social cohesion.^1^

 This paper contributes to this knowledge gap and investigates to what extent social protection measures can influence social cohesion during pandemics.

Social cohesion is a multi-faceted concept, and despite the longstanding literature on social cohesion (Durkheim 1893/1984; Festinger 1950), a universally shared definition is missing (Chan et al. 2006). In this paper, a recent definition of social cohesion is endorsed that identifies three key attributes of social cohesion and their respective measurement—namely *trust, inclusive identity and cooperation*—and two separate dimensions—the *horizontal and the vertical* (Leininger et al. 2021a). The horizontal dimension includes the relationship between individuals or groups within a society, while the vertical dimension refers to the relationship between individuals/groups and state institutions such as the parliament, the police or the courts (see details in “Concept of Social Cohesion” section).

There is mixed evidence on the effects of covariate shocks such as epidemics or pandemics on different attributes of social cohesion. Flueckiger et al. (2019) focus on the Ebola outbreak in West Africa from 2013 to 2016 and show that state legitimacy—proxied by trust in central government (parliament and police)—increased disproportionately in regions with higher exposure to the epidemics and where governments responded more successfully to the epidemic. Aassve et al. (2021) study the impact of the 1918–1919 Spanish flu pandemic on social trust. Analysing the General Social Survey for the United States, they find that individuals whose families emigrated to the United States from a country with many Spanish flu victims display less trust in other people. Aksoy et al. (2020) show that epidemic exposure has a persistent negative effect on trust in political institutions. This effect is larger for individuals who experienced epidemics under weak governments. Borkowska and Laurence (2021) explores the impact of the COVID-19 pandemic on social cohesion in local communities in the UK. They show that the overall levels of social cohesion are lower in periods of the pandemic compared to all of the examined pre-pandemic periods. The decline of social cohesion is particularly high in the most deprived communities and among certain ethnic minority groups.

In general, there is also mixed evidence that social protection can affect different dimensions of social cohesion. Studies show a positive relationship between social protection and dimensions of social cohesion, such as horizontal trust (Adato 2000; Pavanello et al. 2016), horizontal cooperation (Attanasio et al. 2009, 2015) and vertical trust (Evans et al. 2019). Other studies, in contrast, find negative effects on social cohesion in particular on the horizontal dimension that includes social relations between those that have received benefits and those that have not (Hochfeld and Plagerson 2011; Molyneux et al. 2016; Roelen 2017). In addition, negative effects can be found on the societal perceptions of governments (Aytaç 2014; Bruhn 1996; Guo 2009). Another part of the literature that is related to social cohesion is looking at the negative effects of social protection schemes on risk sharing within informal transfer networks. These so-called crowding-out effects of social protection show that beneficiaries invest less in social relations by reducing their contributions to transfer networks (Lenel and Steiner 2020; Strupat and Klohn 2018; Cecchi et al. 2016). However, some studies also detect crowding-in effects meaning that social protections schemes improves social relations (Kang 2004; Takahashi et al. 2019). One of the points emerging from the literature is that a single social protection scheme alone is less likely to accomplish broader objectives, such as social cohesion. Social protection schemes coordinated in a systemic and universal way may provide larger effects.

So far, no study has analysed the relationship between social protection and social cohesion in the presence of a covariate shock, such as a pandemic. Kenya is an ideal setting in which to examine this relationship. Over the past 10 years, the Kenyan social protection sector has evolved and expanded into a social protection system. The 2011 National Social Protection Policy (NSPP) introduced a vision of increasing coverage, improving coordination and bringing about greater *integration* of programmes and services (Government of Kenya 2011). Spending on social protection has grown slightly as a percentage of GDP, increasing from 0.38% in 2017 to 0.45% of GDP in 2019 (World Bank 2019). The Kenyan government has responded to the pandemic by continuing and adapting their two national social assistance programmes: the National Safety Net Programme (NSNP) and the Hunger Safety Net Programme (HSNP) (Doyle and Ikutwa 2021). Beneficiaries of the programmes received lump-sum payments and cash top-ups to the regular cash transfers (see “Social Protection in Kenya” section for more details on the adaptation). Both flagship programmes cover 1.23 million vulnerable households working in the informal economy (Government of Kenya 2017). Kenya was severely impacted by the first wave of the pandemic and the government has established one of the most stringent lockdowns among Sub-Saharan African countries (Hale et al. 2020; Leininger et al. 2021b). In response to the regional variation in the pandemic outbreak, the government imposed different lockdown policies that varied between the counties. For example, movement in and out of some counties, known as the “lockdown counties”, was curtailed for several months, while this policy was not implemented in other counties. These policies partly followed the detection of positive COVID-19 cases, where most of them were detected in Nairobi and Mombasa at the beginning of the pandemic (Ministry of Health – Kenya 2020).

In order to examine the relationship between social protection and social cohesion in this context, this study uses unique primary data from two nationally representative, in-person surveys that were conducted more than one year before and six months after the first wave of the pandemic in Kenya. These repeated cross-sectional surveys include in total 3796 randomly selected households and were realised as a joint project between the Friedrich–Ebert–Stiftung (FES), the International Labour Office (ILO) and the German Development Institute (DIE).^2^ The surveys are representative of the entire informal economy,^3^ which covers the majority of the Kenyan population, including households that receive benefits from the NSNP and HSNP.

Using both cross-sectional surveys allows for the application of a difference-in-differences approach. As the NSNP and the HSNP have been continued during the pandemic and targeting criteria have not been changed, one can compare households that are covered and not covered by these social assistance programmes before and after the first wave of the pandemic.^4^ Furthermore, a heterogeneity analysis has been conducted in order to check whether the effects of social assistance coverage on social cohesion differ between lockdown and non-lockdown regions.

The paper is organised as follows. “Concept of Social Cohesion and Theoretical Considerations” section briefly presents the endorsed concept of social cohesion and highlights the theoretical relationship between social protection and social cohesion in times of covariate shocks. “Spread of COVID-19 and Lockdown Policies” section describes the spread of COVID-19 and the lockdown policies in Kenya. “Social Protection in Kenya” section presents the national social assistance programmes and describes how they have been adapted during the pandemic in Kenya. “Data and Research Design” section introduces the dataset and the definition of the outcome variables that approximate different attributes of social cohesion and presents the econometric model and the robustness checks. “Results” section shows the estimation results, and “Conclusion” section concludes.

## Concept of Social Cohesion and Theoretical Considerations

### Concept of Social Cohesion

This paper endorses the social cohesion definition provided by Leininger et al. (2021a): “social cohesion refers to both the *vertical and the horizontal* relations among members of society and the state as characterised by a set of attitudes and norms that includes *trust, an inclusive identity and cooperation for the common good*”. This narrow concept of social cohesion includes the essential attributes of social cohesion, which are frequently referred to in the literature (Chan et al. 2006; Fonseca et al. 2019; Schiefer and van der Noll 2016). Such a narrow understanding of social cohesion avoids including potential drivers such as inequality or conflicts. Using this concept is favourable in the context of covariate shocks as the presence of such shocks may contribute to an increase in inequality or a higher prevalence of conflicts. So, the concept does not prevent the study of whether and how increasing inequality in times of shocks could impact social cohesion and to what extent social protection schemes can mitigate this effect.

The definition includes three attributes, each of them examined in both dimensions, horizontal and vertical. Following Leininger et al. (2021a) and Burchi et al. (2020), the attributes are as follows.

1. *Trust*
  Trust is an important component of social cohesion (Chan et al. 2006; Dragolov et al. 2013; Langer et al. 2017; Schiefer and van der Noll 2017. One can differentiate between two types of trust: social trust and institutional trust (Langer et al. 2017; Zerfu et al. 2009). Social trust is the “ability to trust people outside one’s familiar or kinship circles” (Mattes and Moreno 2018). It also could act as the “bond that people share across a society and across economic and ethnic groups, religions, and races” (Rothstein and Uslaner 2005). This is the type of trust capturing the horizontal dimension. Institutional trust is the trust towards “formal, legal institutions of the state” such as the parliament, courts or the police (Mattes and Moreno 2018), and refers to the vertical level.
2. *Inclusive identity*
  Individuals have several identities, some superimposed and some freely chosen. A socially cohesive society is one in which individuals with different identities can co-exist in a peaceful way and where certain identities are not dominant over the collective identity. In other words, different group identities are tolerated, recognised and protected. However, in order for a society to be cohesive, it is necessary that people feel first of all part of a broader entity (e.g. the nation) that is more than the sum of individuals and that bridges different identities of a society.
3. *Cooperation for the common good*
  “Cooperation” refers to the positive social interactions within society, while “the common good” refers to the conception of the material and immaterial living conditions of a collectivity. A society in which many people and groups cooperate for interests that go beyond individual interests (van Oorschot and Komter 1998) is considered to have a high level of social cohesion. While the importance of cooperation among individuals and groups (horizontal dimension) has been stressed in the past, this definition also incorporates vertical cooperation (Chan et al. 2006). Individuals cooperate with the state through participation in public life and civic engagement (Acket et al. 2011; Chan et al. 2006; Jenson 2010; Schiefer and van der Noll 2017).

### Theoretical Considerations

Theoretically, social protection can affect social cohesion by helping beneficiaries to cope with covariate shocks. Social protection schemes can prevent beneficiaries from having to sell assets or engage in other costly strategies to deal with covariate shocks. Thereby, beneficiaries can still invest in their livelihoods and may achieve more equal opportunities, which they would not have achieved without social protection. The literature on societies’ resilience capacities in times of large covariate shocks also highlights social protection schemes and their adaptation as important factors (Gerard et al. 2020; Ulrichs et al. 2019). Béné et al. (2012) analysed the overlaps between the key functions of social protection (protect, prevent, promote and transform (Devereux and Sabates-Wheeler 2004)) and the three resilience capacities (absorptive, adaptive and transformative). They found that protective social protection measures, such as social assistance, are the bedrock on which to build absorptive capacity, which allows people to absorb shocks and prevent an immediate increase of poverty. In particular, this supports those that depend on daily earnings or transfers for survival in the informal economy and have difficulty accessing credit.

The described protective effect of social protection might improve attributes of social cohesion such as *institutional trust,* as beneficiaries experience that the state cares about their needs by maintaining and adapting social protection schemes in times of covariate shocks. If states have national social protection schemes in place that can be used as a *national* response to the covariate shock, the protective effect might also impact *inclusive identity* as beneficiaries feel part of a broader entity (e.g. the nation) that is more than the sum of individuals. Further, more equal opportunities and the feeling that one is not neglected can improve *social trust* and *horizontal cooperation*, as beneficiaries realise that members of other societal groups are as much deprived due to the covariate shock as themselves and, therefore, benefit from the schemes in the same way.

However, the responsiveness and adaptation capability of social protection schemes in times of shocks is crucial. Lack of transparency in the adaptation of the scheme and targeting of the beneficiaries, for example, can create feelings of unfairness and resentment, and, thus, worsen social relations (Molyneux et al. 2016). Examples from Cook Islands (United Nations Children's Fund 2021a), Mongolia (United Nations Children's Fund 2021b) and Thailand (United Nations Children's Fund 2021c) demonstrate how universalist approaches to shock-responsive social protection without targeting were key in strengthening the relationship between citizens and the state in the context of COVID-19.

In addition, the adequacy of social protection benefits, that is, the size of the social protection benefits, is important in order to offset or at least mitigate the negative economic and social effects due to the covariate shock. Examples from early responses to the first and second waves of the COVID-19 pandemic suggest that, although benefits were dispersed to those identified as poor, they were often not sufficient to offset additional costs incurred due to the pandemic (Lowe et al. 2021). If the adequacy of social protection benefits are small during times of covariate shocks, it is more difficult to affect attributes of social cohesion.

A further important factor is that governments must highlight that the state plays a key role in the financing and/or management of social protection programmes in times of shocks. Beneficiaries can take that as a signal that the state cares about their interests, which in turn can increase trust in public institutions (Burchi et al. 2020). When social protection measures are communicated as a response of national unity to deal with the shock, it may also improve the beneficiaries feeling of belonging (*inclusive identity*). However, citizens often have limited information about who is financing and/or implementing a social protection scheme. Consequently, there is the possibility that an effective programme characterised by high national ownership would not lead to an increase in institutional trust if the beneficiaries were unable to associate the programme with the true implementer.

## Spread of COVID-19 and Lockdown Policies

This section presents to what extent Kenya was affected by the COVID-19 pandemic and describes the containment measures implemented by the government.

The first case of COVID-19 was confirmed in Kenya on 13 March 2020, and between then and November 2021, more than 254,541 cases and 5325 deaths have been confirmed (or 9.7 deaths per 100,000 people). While COVID-19 cases have been confirmed across the country, in the early stages of the outbreak more than 82% of the COVID-19 cases were found in Nairobi and 14% in the coastal regions of Mombassa, Kwale and Kilifi (World Bank 2020).

**Fig. 1:**
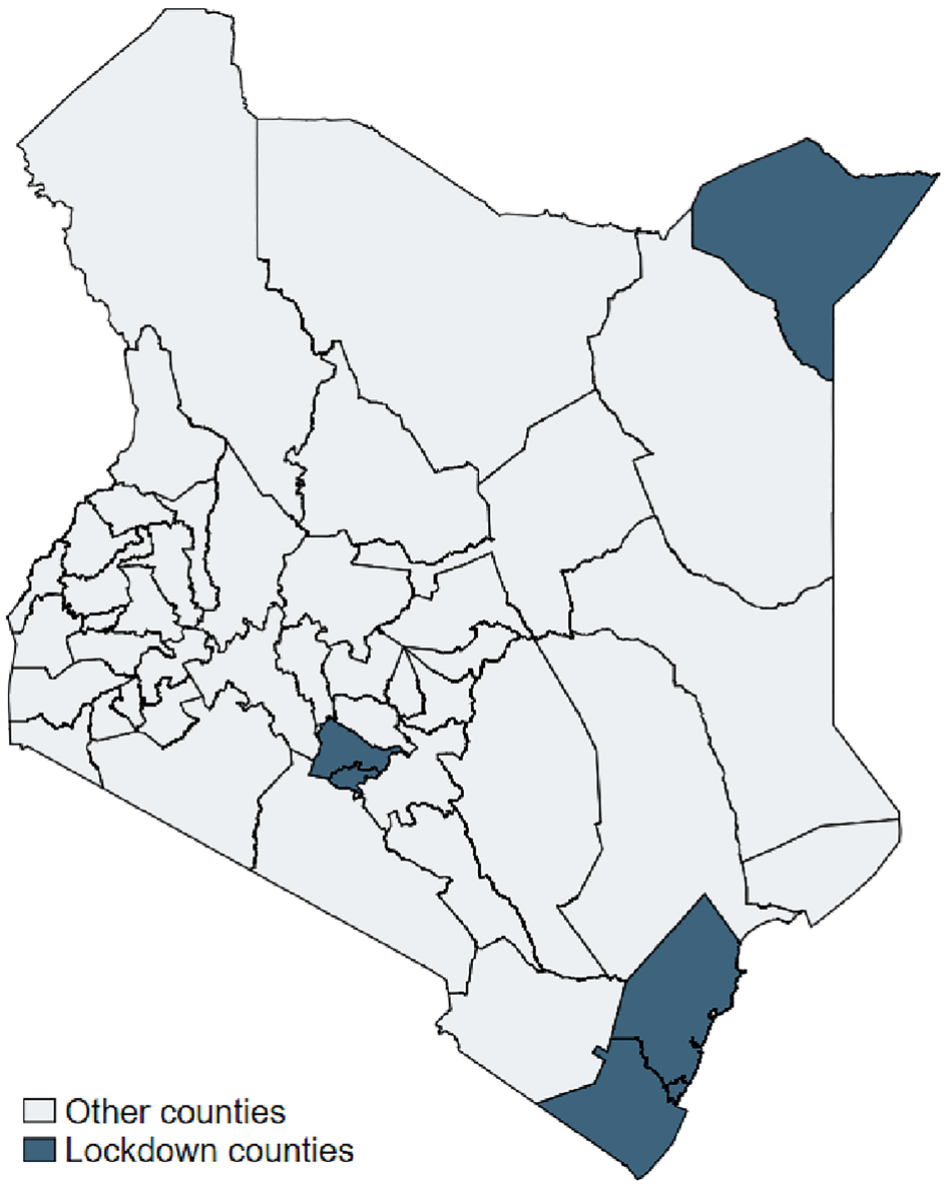
Location of lockdown counties. *Source* Author

In response to the outbreak, on 15 March 2020, the Government of Kenya declared a state of emergency and implemented a range of containment measures. Movement in and out of the six most affected counties, known as the “lockdown counties”, was curtailed for three months in Kilifi and Kwale and four months in Nairobi, Kiambu, Mombasa and Mandera, and markets, restaurants and eateries were closed (see Fig. 1 for locations of lockdown counties) (Doyle and Ikutwa 2021). Importantly, these specific measures did not include stay-at-home requirements during daytime and were ended at latest in July 2020. Further country-wide measures that were imposed in all 47 counties included instructing non-essential public and private sector workers to work from home; banning large social gatherings, including weddings, church gatherings and congregating at malls; and imposing a nationwide night curfew from 7.00 p.m. to 5.00 a.m. Following this, all schools and learning institutions were closed until October 2020. A ban on international passenger flights lasted until August 2020 (Doyle and Ikutwa 2021).

Kenya’s economy contracted by 0.4% between January and June 2020, a stark contrast with the growth of 5.4% during the same period in 2019 (World Bank 2020a). COVID-19 and the containment measures had the most severe socioeconomic impacts in Nairobi and the other lockdown counties where, initially, cases were highest and lockdown measures were most stringent (World Bank 2021). Country-wide unemployment is almost double what it was before COVID-19, and the labour force participation rate has decreased. Close to half of the informal labour force in the lockdown counties and one-third of it in the other counties had to discontinue their labour activities for almost 12 weeks. Overall, the World Bank (2021) reports that earnings have significantly decreased for wage earners in the informal sector. Moreover, the reduction in earnings was found to be greater for informal workers in the lockdown counties (42%) than in other counties (24%). In addition, COVID-19 is estimated to increase poverty in Kenya by about 4 percentage points resulting in 2 million newly poor Kenyans (World Bank 2020a).

Excessive violence against civilians was used by the police to enforce the lockdown measures in the lockdown counties. Police killed 15 people and injured 31 while the lockdown measures were imposed. There were also numerous arrests of those violating curfew rules (Citizen Reporter 2020). Vendors protested their loss of livelihood due to movement restrictions and the mandated closure of businesses in the lockdown counties. There were brief incidences of social unrest in some areas of Nairobi when the lockdown measures were imposed (Renner 2020). These demonstrations did not lead to mass scale civil unrest, but the government apologised about police brutality against citizens during the protests and curfew hours (Kemboi 2020).

## Social Protection in Kenya

This section briefly presents the social protection system in Kenya and focuses on the description of the national social assistance programmes and how they have been adapted during the COVID-19 pandemic.

Over the past 10 years, the Kenyan social protection sector has evolved and expanded into a social protection system. The 2011 NSPP introduced a vision of increasing coverage, improving coordination and bringing about greater integration of programmes and services (Government of Kenya 2011). Social protection in Kenya is currently structured along the three main pillars of social assistance, social security and health insurance (Government of Kenya 2017).^5^ The most prominent programme under these pillars is the NSNP, which has been adapted during the pandemic. It consists of the Older Persons Cash Transfer (OP-CT), the Cash Transfer for Orphans and Vulnerable Children (CT-OVC) and the Persons with Severe Disabilities Cash Transfer (PWSD-CT). These three cash transfer programmes give beneficiary households a transfer of KES 2,000 (USD 18) per month.^6^ Target households are living in poverty and have at least one household member that falls under the categories covered by each programme (orphans and vulnerable children, elderly and people with severe disabilities). The HSNP is the fourth cash transfer programme; it is implemented by the National Drought Management Authority (NDMA). It targets households that cannot afford to meet basic expenses (regular nutritious food, adequate housing, sanitation, etc.) and are vulnerable to becoming poorer in times of shocks, for example, drought, livestock disease and floods. The programme provides KES 5,400 (USD 50) every two months.^7^ The Government of Kenya directly finances 100% of the four cash transfer programmes, which collectively reach 1.23 million households across all counties (Doyle and Ikutwa 2021).

As a response to the COVID pandemic, the government announced on 25 March 2020 the *continuation of* NSNP/HSNP and that funds previously committed would be released so that the pandemic would not impact the timely delivery of benefits. Consequently, beneficiaries received a lump sum of KES 8000 (USD 74) to cover the period January to April 2020 (two regular payment cycles were pooled). A second tranche of KES 4000 (approx. USD 37) was disbursed as a lump sum at the end of June 2020 to cover May and June 2020 (Doyle and Ikutwa 2021). Vertical expansions that temporarily increased the level of support to NSNP beneficiaries by providing cash top-ups to the regular cash transfers were provided by the United Nations Children’s Fund (UNICEF) and an EU-funded consortium led by the Kenyan Red Cross Society and Oxfam. UNICEF provided two monthly cash top-up payments of KES 2000 per month to all NSNP beneficiaries with children under the age of 10. The EU consortium provided monthly cash top-ups of KES 5668 (approx. USD 52) for three months to all NSNP beneficiaries residing in informal settlements. The continuation and adaptations of the NSNP and HSNP were highlighted in public appeals of the government to “stand together” in order to cope with the pandemic (Government of Kenya 2020).

The government also set up new short-term social assistance programmes to cushion some of the negative socioeconomic consequences of the pandemic. They target households that are not enrolled in the NSNP or HSNP. This short-term response consists of the multi-agency COVID-19 cash transfer and the National Council for Persons with Disabilities (NCPWD) cash transfer. Both programmes target the chronically sick, widowers, the elderly and persons with disabilities. The response took the form of a weekly cash transfer of KES 1000 (approx. USD 10) for a period of three to four months and reached 669,000 households (Doyle and Ikutwa 2021).

## Data and Research Design

### Data

The analysis in the study is based on primary data from 3796 randomly selected households that operate in the informal economy. Between November and December of 2018, 1188 households were surveyed, and in December 2020, after lockdown measures were eased, 2608 households were surveyed. The surveys were realised as a joint project between the FES, the ILO and DIE. The surveys were designed as repeated country-representative cross-sections of households in the informal economy. The data was collected through in-person interviews with the household head and one randomly selected household member over the age of 15.^8^ The main objectives of the surveys were to obtain a better understanding of the economic and social situation of the informal economy before and after the first wave of the COVID pandemic. The questionnaire included modules on household demographics, health, social protection programmes, social cohesion and self-organisations. The selected sample was determined by random selection methods at every stage of sampling and the application of probability sampling was based on population data (see detailed description of the sampling design and sampling process in the “Appendix”).^9^

The present study concentrates on outcomes related to social cohesion. Following the concept of social cohesion (see “Concept of Social Cohesion” section), the questionnaire inquired about the three attributes of social cohesion: trust, inclusive identity and cooperation for a common good.

The first two questions measure trust according to the social cohesion definition used for this paper. The first asks respondents whether at the time of the survey they trusted the parliament and the government. Answers ranged from “not at all” (coded “0”) to “a lot” (coded “3”). This question is used to measure institutional (vertical) trust. Please note that in the social cohesion definition of Leininger et al. (2021a) trust in the government is not part of the measure for institutional trust as the concept aims at measuring trust in institutions. Unfortunately, there are no further measures on institutional trust, such as trust in the police or courts that cover both survey rounds. Additionally, the measure on social trust is not available for both survey rounds due to data limitations.

The second question that was used approximates the attribute “identity” in the social cohesion definition. Respondents were asked about their agreement or disagreement with the following statement: “It makes me proud to be called a Kenyan”. Answers ranged from “strongly disagree” (coded “0”) to “strongly agree” (coded “4”).

The third questions refer to the social cohesion attribute “cooperation”. The question asked respondents: “How often did you do voluntary work with others such as help out with food or cash or doing community work?” The answer options ranged from “never” (coded “0”) to “occasionally (once per month)” (coded “3”) to “very frequently (every day)” (coded “5”). This measure approximates the horizontal dimension of cooperation. In general, to assess cooperation in the Kenyan context including lockdown policies is possible, as the lockdown measures did not include stay-at-home restrictions and the survey was conducted six months after the ease of lockdowns. A measure on vertical cooperation is not available due to data limitations. Questions of institutional trust were transformed to binary indicators so that they take the value “0” if the respondent answered “not at all” or “just a little” and the value “1” if the respondent answered “somewhat” or “a lot”. Similarly, binary variables were created for other question formulations: taking value “0” if the respondent answered “strongly disagree” or “disagree” and value “1” if the answers were “agree” or “strongly agree”. For the question on cooperation, the variable takes the value of “0” if the respondents answered “rarely (3 to 6 times per year)”, “very rarely (1 or 2 times per year)” or “never”, and it takes the value “1” if the respondents answered “occasionally (once per month)”, “frequently (once per week)” or “very frequently (every day)”.

Table 1 shows the means of the four outcome variables for the time before and after the first wave of the pandemic. Lower levels in the social cohesion attributes can be detected for cooperation, trust in the government and trust in the parliament. Trust in the government declines by 4 percentage points, respectively, while cooperation decreases by 4 percentage points. No statistical significant differences can be detected with regards to the attribute of identity and trust in parliament.

**Table 1:** Means of the outcome variables before and after the first wave of the pandemic

	After first wave of pandemic	Before pandemic	Difference
*Outcomes*			
Trust in government	0.80 (0.01)	0.84 (0.01)	− 0.04** (0.01)
Trust in parliament	0.70 (0.01)	0.72 (0.01)	− 0.02 (0.02)
Inclusive identity	0.93 (0.01)	0.93 (0.01)	0.00 (0.01)
Cooperation (horizontal)	0.24 (0.01)	0.28 (0.01)	− 0.04*** (0.01)
*N*	2608	1188	

Standard errors are in parenthesis, *p < 0.10, **p < 0.05, ***p < 0.01

The survey team asked the household head whether the household is covered by the NSNP (including the three cash transfer programmes), the HSNP or any other existing social assistance programme (see “Social Protection in Kenya” section). Enrolment status was checked by the enumerators using either identification documents or the NSNP card. In order to separate existing social assistance programmes from new short-term programmes, the enumerators first asked whether the respondents had received any support in cash since the COVID-19 outbreak. If yes, they were asked if it was received from the national government, the local government or an employer. If it was from the national government, the respondents were asked to indicate the programme from which they received the cash transfers. At the end, they were asked to report the amount of cash they received.

As the focus of the paper is to examine the effects of existing social assistance programmes (such as the NSNP and HSNP) during the pandemic, Table 2 presents the mean coverage of these programmes before and after the first wave of the pandemic. As the government of Kenya managed to minimise disruptions to the routine delivery of benefits, 12% of our sample were covered by the NSNP or HSNP in 2020. This share is in line with the 1.23 million households that were covered by social assistance in 2020, which represent 12% of the 10 million households of the informal sector (KNBS 2019).

**Table 2:** Social assistance coverage before and after the first wave of the pandemic

	After first wave of pandemic	Before pandemic	Difference
Social assistance (NSNP and HSNP)	0.12 (0.01)	0.11 (0.01)	0.01 (0.01)
*N*	2608	1188	

Standard errors are in parenthesis, *p < 0.10, **p < 0.05, ***p < 0.01

### Empirical Specification

The estimation strategy used for this study exploits the effect of the national social assistance programmes (such as the NSNP and HSNP) during the COVID-19 pandemic in a difference-in-differences setting. More specifically, members of households with and without coverage of national social assistance programmes (NSNP and HSNP) are compared before and after the first wave of the pandemic using repeated cross-sectional data.^10^ To employ the difference-in-differences strategy, the following linear regression specification is estimated.

1 $${y}_{ict}={\beta }_{0}+\left({T}_{t}\cdot {SA}_{ict}\right){\beta }_{1}+{T}_{t}{\beta }_{2}+{SA}_{ict}{\beta }_{3}+ {\mathrm{X}}_{ict}{\beta }_{4}+ {\sum }_{c=1}^{47}{\mu }_{c}\left({County}_{c}\right)+{\epsilon }_{ict},$$

$${y}_{ict}$$ represents the outcome of interest (trust in government, trust in parliament, inclusive identity and horizontal cooperation) for respondent *i* residing in county *c* at the time of each survey *t*.^11^ This variable is regressed on the interactions between the binary variable $${T}_{t}$$ which takes the value “1” after the first wave of the pandemic at the end of 2020 and the binary variable $${SA}_{ict}$$ which takes the value “1” if the household of respondent *i* is covered by national social assistance programmes (NSNP or HSNP) at the time of the survey *t*. $${X}_{ict}$$ is a set of individual and household characteristics observed at the time of each survey including age and sex of the respondent, education level of the respondent, marital status of the respondent, chronic illness and disability in the household, household size, gender of the household head, the household’s share of elderly and children, and coverage from other social protection measures, such as the new short-term social assistance programmes or a health insurance scheme.^12^ In order to account for the different initial development levels of the counties that are possibly related to the outcome variables and social assistance coverage, 47 county dummies ($${County}_{c})$$ are included. They control for the time-invariant unobserved heterogeneity between the counties. $${\epsilon }_{ict}$$ is the usual error term.

The coefficients of interest are ${\beta }_{1}$ and ${ \beta }_{2}$.${\beta }_{1}$ measures the effect of the national social assistance coverage after the first wave of the pandemic on the outcome variables. ${\beta }_{2}$ shows the effect of the first wave of the pandemic on those that are not covered by social assistance. Whether one can interpret these effects as causal depends critically on the identifying assumption. Conditional on the controls included in Specification (1), the identifying assumption is that respondents with and without coverage of the national social assistance programmes would have had the same time trend in the selected outcome variables if the national social assistance programmes would not have been continued during the pandemic. Because the national social assistance programmes (NSNP and HSNP) have been continued/adapted by the government during the pandemic, this assumption is not directly testable. In order to check if this parallel trend assumption potentially holds one normally resorts to data before the pandemic. Unfortunately, the employed data consists only of two survey rounds with one pre-pandemic data point, which makes it not possible to test for parallel trends of outcomes between respondents with and without coverage of the national social assistance programmes before the pandemic. A causal interpretation of the results is therefore not warranted.

To alleviate some concerns that the results of the difference-in-differences model (specification 1) are entirely driven by changes in other underlying factors, three robustness checks have been conducted.^13^ A first check of robustness consists of including interactions between the controls *X* and the survey round indicator *T* to take into account the possibility that these variables had a differential impact on social cohesion in the period after the first wave of the pandemic. If the results do not change this would indicate that changes in the outcomes are not due to changes in other underlying factors or compositional changes of the samples (see “Empirical Results” section for results). This check is of importance when using repeated cross-sectional data (La Ferrara and Milazzo 2017).

In addition, to consider that the pandemic has affected counties differently over time, which also can explain changes in social cohesion, county-specific time trends have been added to the regression specification (1). So it is possible to consider county-specific changes between the first and second round of the survey as robustness check. These can be done by interacting the 47 county dummy variables ($${County}_{c})$$ with the survey round indicator *T*. If the results remain similar after considering these time-variant heterogeneity between the counties, this would indicate that the findings are not driven by county-specific changes due to the pandemic.

The final check for robustness extends the difference-in-differences model (specification 1) by a kernel propensity-score matching (Villa 2016) on both rounds of the repeated cross-sectional surveys (following Blundell and Costa Dias (2009)). This allows for the control group to be matched to the social assistance beneficiaries using the individual and household characteristics (see more details in “Robustness Checks” section).

### Heterogeneity Analysis Using Lockdown Counties

In order to explore whether the effect of social assistance is heterogeneous between lockdown and non-lockdown counties, the main Specification (1) is adapted and the following triple-differences model is estimated.

2 $${y}_{ict}={\beta }_{0}+\left({T}_{t}\cdot {SA}_{ict}\cdot {L}_{c}\right){\beta }_{1}+\left({T}_{t}\cdot {L}_{c}\right){\beta }_{2}+\left({T}_{t}\cdot {SA}_{ict}\right){\beta }_{3}+\left({SA}_{ict}\cdot {L}_{c}\right){\beta }_{4}+{T}_{t}{\beta }_{5}+{SA}_{ict}{\beta }_{6}+ {\gamma }^{\mathrm{^{\prime}}}{\mathrm{X}}_{ict}+ {\sum }_{c=1}^{47}{\mu }_{c}\left({County}_{c}\right)+{\epsilon }_{ict},$$

${L}_{c}$ represents a binary indicator that takes the value “1” if respondents reside in the six lockdown counties (see Fig. 1). The triple-differences model follows Cunningham (2021). It includes each variable independently, each individual interaction and the triple-differences interaction. As the inclusion of the county dummies considers all county-specific time-invariant factors, the variable ${L}_{c}$ (without interaction) was omitted by the model. In this specification $,{ \beta }_{1}$ measures the effect of national social assistance coverage in lockdown counties compared with non-lockdown counties after the first wave of the pandemic on the outcome variables. ${\beta }_{2}$ shows the effect of the first wave of the pandemic on those that reside in lockdown counties compared with those in non-lockdown counties who are not covered by social assistance.

Similar with specification (1), due to data limitations, it is unfortunately not possible to check if the parallel trends assumption holds and outcomes of respondents with and without social assistance coverage for lockdown and non-lockdown counties had the same trend before the pandemic. Additionally, time-varying unobservable factors that might only affect social assistance beneficiaries in lockdown counties can confound the triple-differences estimates. For example, if unobservable changes in social cohesion only affect social assistance beneficiaries in lockdown counties, but do not affect them in non-lockdown counties. So the results of this heterogeneity analysis should be interpreted with caution, as they might be affected by time-variant unobservable factors.

## Results

### Descriptive Results

Table 3 shows the means of the four outcome variables for the two groups across the two survey rounds. It seems that before the pandemic there were no statistically significant differences in levels of social cohesion between those with and without social assistance. The difference-in-differences reveal an increase in trust in government and horizontal cooperation by 5 and 6 percentage points (see Column 3). The double difference also shows that households that were covered by regular social assistance exhibit a higher likelihood of trusting the parliament and higher inclusive identity, but the effects are not statistically significant. It seems that households that do not receive regular social assistance experience a decrease in institutional trust and cooperation. However, it is important to consider individual/household characteristics and the set of county dummies in order to control for confounding factors, so the next subsection gives the results of the econometric model.

**Table 3:** Means of the outcome variables by social assistance coverage before and after the first wave of the pandemic

	After first wave of pandemic			Before pandemic		
	1	2	3	4	5	6
	Social assistance	No social assistance	Double diff. (1–2) − (4–5)	Social assistance	No social assistance	Single diff. (4 − 5)
*Outcomes*						
Trust in government	0.89 (0.02)	0.81 (0.01)	0.05** (0.03)	0.87 (0.01)	0.84 (0.01)	0.03 (0.03)
Trust in parliament	0.77 (0.03)	0.70 (0.01)	0.02 (0.05)	0.77 (0.04)	0.72 (0.01)	0.05 (0.04)
Inclusive identity	0.95 (0.01)	0.93 (0.01)	0.03 (0.03)	0.92 (0.02)	0.93 (0.01)	− 0.01 (0.02)
Cooperation	0.29 (0.01)	0.24 (0.01)	0.06** (0.01)	0.27 (0.03)	0.28 (0.01)	− 0.01 (0.01)
*N*	313	2426		125	1063	

Standard errors are in parenthesis, *p < 0.10, **p < 0.05, ***p < 0.01

### Empirical Results

Table 4 reports the estimation results from the main econometric specification (Specification (1)) for the four outcome variables illustrated in “Data and Research Design” section (see Table 9 of the Appendix for full results).^14^ The signs of the estimated coefficients (${\beta }_{1})$ are positive; however, the effects are not statistically significant. Turning to the coefficients for those that are not covered by social assistance after the first wave of the pandemic (${\beta }_{2})$ reveal a significant decrease of social cohesion attributes. Trust in government is reduced by 4 percentage points, while the willingness to cooperate with others to do voluntary work is reduced by 2 percentage points. The findings suggest that social assistance has no effect on attributes of social cohesion in times of the pandemic.

**Table 4:** Effects of social assistance on attributes of social cohesion

Outcome variables	Trust parliament	Trust government	Inclusive Identity	Cooperation (horizontal)
(${\beta }_{1})$ Social assistance*After first wave of pandemic	0.05 (0.06)	0.04 (0.04)	0.05 (0.04)	0.01 (0.01)
(${\beta }_{2})$ After first wave of pandemic	− 0.03 (0.02)	− 0.04** (0.02)	− 0.01 (0.01)	− 0.02** (0.01)
(${\beta }_{3})$ Social assistance	0.04 (0.05)	0.03 (0.03)	− 0.02 (0.03)	0.01 (0.01)
*N*	3416	3416	3416	3416
adj. R-sq	0.02	0.02	0.01	0.03

Control variables and county dummies are included. Standard errors (in parenthesis) are clustered at the county level, *p < 0.10, **p < 0.05, ***p < 0.01

Interestingly, if one focuses on the heterogeneity of the social assistance effect between lockdown and non-lockdown counties (see Specification (2) in “Data and Research Design” section), one finds a positive and statistically significant effect of social assistance on trust in government and horizontal cooperation. Table 5 shows that social assistance coverage in lockdown counties improves trust in the government by 3 percentages points, which is a relative increase of 4%. Furthermore, the willingness to cooperate with others to do voluntary work, such as help others with food or cash increases by 4 percentage points, which is a relative increase of 16% (see Table 10 of the Appendix for full results).^15^ No statistically significant effects can be detected for the other attributes of social cohesion. However, the signs of the estimated coefficients are positive. It is unclear whether the cooperation effect is related to joint activities to help others with food or cash so that they can cope with the negative consequences of lockdowns.^16^ Nevertheless, as mentioned above the results of this heterogeneity analysis should be interpreted with caution, as they might be affected by time-variant unobservable factors.

### Robustness Checks

In order to check whether the explanatory variables have a differential impact on social cohesion after the first wave of the pandemic, interaction terms between the controls *X* and the survey round indicator *T* were included in regression specification (1). Results are presented in Table 11 of the Appendix. The estimates of interests remain similar after the inclusion of the interaction terms, suggesting that the effects on social cohesion are due to the national social assistance programmes and not due to compositional changes of the samples and the differential impact of the control variables over time.

An additional check of robustness includes whether the results do change if one considers county-specific time trends. Interaction terms between the 47 county dummy variables ($${County}_{c})$$ and the survey round indicator *T* were included in regression specification (1). The estimates of interests remain similar after the inclusion of the interaction terms, suggesting that the effects on social cohesion are not driven by county-specific changes due to the pandemic. Tables 12 of the Appendix show the estimation results. The results remain similar to the original difference-in-differences approach.

The last check of robustness extends the difference-in-differences model (specification 1) by a kernel propensity-score matching (Villa 2016) on both rounds of the repeated cross-sectional surveys. The PSM-kernel matching was conducted by using the individual and household characteristics of specification (1). The common support is composed of the social assistance beneficiaries to whom a counterfactual is found in the control group sample. As the estimation of this robustness check is limited to the common support, the number of observations drops from 3416 to 2992. The Tables 13 and 14 show the weighted means after applying the PSM-kernel matching for both groups (balancing tests) on all observable characteristics before the pandemic and after the first wave of the pandemic. This approach increases the homogeneity of those with and without coverage of national social assistance programmes in terms of observable characteristics and could also raise the similarity in unobserved characteristics. After balancing on the covariates, the 47 county dummies were included in the estimation model. Table 15 of the Appendix shows the estimation results. The results remain similar to the original difference-in-differences approach.

## Conclusion

As it is unclear whether social assistance measures affect social cohesion in times of large covariate shocks such as a pandemic, this study attempts to close this knowledge gap by focusing on the relationship between social assistance and social cohesion in Kenya during the COVID-19 pandemic. The continuation and adaptation of existing social assistance programmes in response to the COVID-19 pandemic, coupled with regional differences in impacts of the pandemic and lockdown policies, makes Kenya an ideal setting for examining this relationship.^17^ The analysis is based on a difference-in-differences approach and unique primary data from repeated country-representative in-person surveys that were collected more than one year before and six months after the first wave of the pandemic.

The main findings suggest that social assistance does not influence attributes of social cohesion in times of the pandemic. As highlighted in the theory section, one potential explanation of this finding is that social assistance benefits might be in general too small to entirely offset the negative economic consequences of the pandemic so that the potential protective effect of social protection on social cohesion in times of large covariate shocks could not become relevant. The Kenya Cash Working Group (KCWG) recommends that cash transfers provide a minimum level of support equivalent to 50% of the minimum expenditure basket of the household for three months. The social assistance benefits covered only 25% of the expenditure basket during the first phase of the pandemic (Doyle and Ikutwa 2021) and the adaption was not adequate to prevent all negative economic impacts of the pandemic. In general, this finding is in line with studies showing that cash benefit programmes which are not adequately designed and implemented do not necessarily increase social cohesion (Li and Walker 2017; Roelen 2017; Burchi and Roscioli 2021). Policy makers who want to strengthen social cohesion should be aware of the limitations of cash-only social assistance programmes and should consider a more adequate adaption of such programmes in times of pandemics. Focusing on the heterogeneity of the social assistance effect between lockdown and non-lockdown counties one finds a positive and statistically significant effect of social assistance on trust in government and horizontal cooperation for lockdown counties. However, the results of this heterogeneity analysis should be interpreted with caution, as they might be biased by time-variant confounders.

A general remark is necessary. From a methodological point of view, the caveat of the analysis is of course the missing plausibility test of the parallel trends assumption. The results might be due to diverging trends in the social cohesion outcomes between social assistance and non-social assistance beneficiaries before the pandemic. The used unique primary data covering indicators of social cohesion and social protection within the informal economy, does not allow to resort to rounds of data before the pandemic. Therefore, the results should be interpreted with caution and no causal interpretation is warranted. As no study so far has analysed the relationship between social protection and social cohesion in the presence of a covariate shock, such as a pandemic, this study should be seen as first step and a promising avenue for future research would therefore be to examine the same relationship using several rounds of pre-pandemic data from different country contexts. This analysis should also take into account all the different aspects of the complex concept of social cohesion. In particular, the analysis should concentrate on all dimensions of social cohesion according to the employed concept (see “Concept of Social Cohesion” section). This would give a more detailed and complete picture of the effects of social protection on social cohesion during times of covariate shocks.

## Appendix

See Tables 6, 7, 8, 9, 10, 11, 12, 13, 14, and 15.

**Table 6:** Means of the explanatory variables

	After first wave of pandemic	Before pandemic	Difference	Std. Error
Social assistance coverage (NSNP or HSNP)	0.1229	0.1091	− 0.0138	0.0103
Age 15–29	0.3443	0.3677	− 0.0234	0.0176
Age 30–39	0.2534	0.2616	− 0.0082	0.0165
Age 40–49	0.1959	0.1868	0.0091	0.0148
Age 50–49	0.1205	0.1091	0.0113	0.012
Age > 60	0.0759	0.0747	0.0012	0.011
Female	0.5265	0.5285	− 0.0020	0.0187
No education	0.095	0.0855	0.0095	0.0108
Primary education	0.5732	0.5641	0.0091	0.0185
Secondary education	0.3022	0.297	0.0053	0.0172
University education	0.0296	0.0334	− 0.0038	0.0065
Married	0.6832	0.6942	− 0.011	0.0174
Disability in the household	0.0679	0.0875	− 0.0196**	0.0098
Chronic illness in the household	0.1167	0.1485	− 0.0318***	0.0121
Household size	4.4019	4.2829	0.1190	0.0890
Share of elderly (age > 60) in household	0.0629	0.0574	0.0055	0.0068
Share of children (age < 15) in household	0.3119	0.3095	0.0024	0.0093
Short-term social assistance (COVID related)	0.0863	0	0.0863***	0.0083
Health insurance (household)	0.2626	0.2575	0.0051	0.0161
Number of observations	2399	1017		

*, ** and *** denote *p* < 0.10, *p* < 0.05 and *p* < 0.01, respectively

**Table 7:** Means of the explanatory variables by social assistance coverage *before* the pandemic

	Social assistance	No social assistance	Difference	Std. Error
Age 15–29	0.384	0.3744	0.0096	0.0458
Age 30–39	0.228	0.2747	− 0.0467	0.0421
Age 40–49	0.201	0.1797	0.0203	0.0365
Age 50–49	0.112	0.1016	0.0104	0.0287
Age > 60	0.076	0.0696	0.0064	0.0239
Female	0.544	0.5278	0.0162	0.0472
No education	0.162	0.0706	0.0914***	0.0254
Primary education	0.576	0.556	0.0200	0.047
Secondary education	0.256	0.3283	− 0.0723	0.0441
University education	0.010	0.0452	− 0.0352*	0.019
Married	0.680	0.7159	− 0.0359	0.0428
Disability in the household	0.104	0.0681	0.0359	0.0258
Chronic illness in the household	0.224	0.1373	0.0867***	0.0334
Household size	4.344	4.2333	0.1107	0.2063
Share of elderly (age > 60) in household	0.0675	0.0512	0.0163	0.0156
Share of children (age < 15) in household	0.3382	0.3164	0.0218	0.0232
Health insurance (household)	0.2480	0.2568	0.0088	0.0413
Number of observations	111	906		

*, ** and *** denote *p* < 0.10, *p* < 0.05 and *p* < 0.01, respectively

**Table 8:** Means of the explanatory variables by social assistance coverage *after* the first wave of the pandemic

	Social assistance	No social assistance	Difference	Std. Error
Age 15–29	0.3429	0.3415	0.0014	0.0364
Age 30–39	0.2171	0.2671	− 0.0499	0.0346
Age 40–49	0.2071	0.1911	0.016	0.0311
Age 50–49	0.1429	0.1187	0.0242	0.0256
Age > 60	0.0901	0.0716	0.0185	0.0241
Female	0.5286	0.5184	0.0102	0.0392
No education	0.1901	0.0868	0.1032***	0.0229
Primary education	0.5586	0.5751	− 0.0165	0.0388
Secondary education	0.2343	0.3076	− 0.0733**	0.036
University education	0.0101	0.0306	− 0.0205	0.0133
Married	0.6286	0.6875	− 0.0589	0.0365
Disability in the household	0.1043	0.0674	0.0369*	0.0198
Chronic illness in the household	0.1714	0.1116	0.0598***	0.0242
Household size	4.5643	4.3942	0.1701	0.1912
Share of elderly (age > 60) in household	0.0753	0.0619	0.0134	0.0146
Share of children (age < 15) in household	0.2978	0.3146	− 0.0168	0.0196
Short-term social assistance (COVID related)	0.0468	0.0454	0.0014	0.0190
Health insurance (household)	0.2401	0.2644	0.0244	0.0346
Number of observations	288	2111		

*, ** and *** denote *p* < 0.10, *p* < 0.05 and *p* < 0.01, respectively

**Table 9:** Effects of social assistance on attributes of social cohesion

	(1)	(2)	(3)	(4)
	Inclusive identity	Cooperation (horizontal)	Trust government	Trust parliament
Social assistance*After first wave of pandemic	0.0480 (0.0379)	0.00619 (0.0106)	0.0443 (0.0438)	0.0494 (0.0563)
After first wave of pandemic	− 0.0106 (0.0101)	− 0.0250*** (0.00867)	− 0.0378** (0.0191)	− 0.0110 (0.0203)
Social assistance	− 0.0205 (0.0345)	0.00794 (0.0106)	0.0338 (0.0327)	0.0422 (0.0484)
Age 30–39	− 0.0206 (0.0123)	− 0.00838 (0.0130)	− 0.0427*** (0.0128)	− 0.0462*** (0.0145)
Age 40–49	0.00334 (0.0148)	0.00595 (0.0184)	0.00181 (0.0178)	− 0.0417* (0.0245)
Age 50–59	− 0.0104 (0.0173)	− 0.00763 (0.0234)	− 0.0117 (0.0217)	− 0.0273 (0.0263)
Age > 60	0.00869 (0.0193)	0.00641 (0.00397)	0.0105 (0.0362)	0.0314 (0.0426)
Female	0.0394*** (0.00872)	− 0.0106 (0.0102)	− 0.00950 (0.0125)	0.0184 (0.0157)
Primary education	0.0436*** (0.0137)	0.0337 (0.0279)	0.0225 (0.0284)	0.0160 (0.0359)
Secondary education	0.0220 (0.0173)	0.0257 (0.0298)	− 0.0177 (0.0321)	− 0.0403 (0.0368)
University education	− 0.0500 (0.0387)	− 0.0447 (0.0293)	− 0.0360 (0.0436)	− 0.0358 (0.0487)
Married	− 0.000920 (0.00910)	− 0.0191* (0.0106)	0.0233 (0.0164)	0.0346** (0.0167)
Disability in household	− 0.0377 (0.0323)	0.00770 (0.0210)	− 0.00674 (0.0235)	− 0.0473 (0.0295)
Chronic illness in household	− 0.0162 (0.0142)	− 0.0206 (0.0189)	− 0.0424** (0.0192)	− 0.0587*** (0.0215)
Household size	0.00309* (0.00176)	− 0.00381 (0.00292)	− 0.00402 (0.00290)	− 0.0119*** (0.00374)
Share of children in household	0.0155 (0.0171)	0.0542** (0.0265)	0.0499 (0.0302)	0.0503 (0.0375)
Share of elderly (age > 60) in household	0.0445* (0.0264)	− 0.0109 (0.00804)	0.0252 (0.0568)	− 0.0341 (0.0647)
Short-term social assistance (COVID related)	− 0.00839 (0.0261)	− 0.00130 (0.00151)	0.0378 (0.0383)	− 0.00392 (0.0458)
Health insurance (household)	0.00235 (0.00993)	− 0.00241 (0.00186)	− 0.0314* (0.0158)	− 0.0340* (0.0195)
Observations	3416	3416	3416	3416
R-squared	0.020	0.027	0.015	0.015

County dummies are included. Standard errors (in parentheses) are clustered at the county level, **p* < 0.10, ***p* < 0.05, ****p* < 0.01

**Table 10:** Heterogeneity of social assistance effects due to differences in regional lockdown policies

	(1)	(2)	(3)	(4)
	Inclusive identity	Cooperation (horizontal)	Trust government	Trust parliament
Social assistance*After first wave of pandemic*Lockdown county	0.00947 (0.0669)	0.0376** (0.0159)	0.0319** (0.0137)	0.026 (0.0950)
After first wave of pandemic*Lockdown county	− 0.0542*** (0.0150)	− 0.0189 (0.0185)	− 0.0448* (0.0265)	− 0.0969*** (0.0320)
After first wave of pandemic*Social assistance	0.0340 (0.0474)	0.0135 (0.0128)	0.0177 (0.0560)	0.00417 (0.0698)
Social assistance*Lockdown county	0.0403 (0.0605)	− 0.0156 (0.0159)	− 0.0447 (0.0707)	− 0.0133 (0.0925)
After first wave of pandemic	− 0.0227* (0.0113)	− 0.0250** (0.0102)	− 0.0212* (0.0118)	0.00281 (0.0231)
Social assistance	− 0.0280 (0.0409)	0.0142 (0.0124)	0.0491 (0.0389)	0.0729 (0.0456)
Age 30–39	− 0.0209* (0.0124)	− 0.0145 (0.0130)	− 0.0457*** (0.0128)	− 0.0453*** (0.0147)
Age 40–49	0.00342 (0.0148)	0.000145 (0.0220)	0.00227 (0.0178)	− 0.0424* (0.0250)
Age 50–59	− 0.0112 (0.0174)	− 0.0111 (0.00231)	− 0.00934 (0.0216)	− 0.0278 (0.0264)
Age > 60	0.00711 (0.0194)	0.00644 (0.00460)	0.0139 (0.0363)	0.0323 (0.0427)
Female	0.0395*** (0.00881)	− 0.0104 (0.00988)	− 0.00992 (0.0123)	0.0186 (0.0159)
Primary education	0.0438*** (0.0139)	0.0335 (0.0270)	0.0225 (0.0283)	0.0158 (0.0359)
Secondary education	0.0212 (0.0172)	0.0238 (0.0291)	− 0.0176 (0.0324)	− 0.0370 (0.0369)
University education	− 0.0529 (0.0385)	− 0.0480 (0.0383)	− 0.0326 (0.0437)	− 0.0313 (0.0486)
Married	− 0.000979 (0.00900)	− 0.0195* (0.0108)	0.0225 (0.0165)	0.0359** (0.0170)
Disability in the household	− 0.0382 (0.0326)	0.00660 (0.0207)	− 0.00555 (0.0235)	− 0.0470 (0.0299)
Chronic illness in the household	− 0.0165 (0.0143)	− 0.0242 (0.0197)	− 0.0443** (0.0195)	− 0.0550** (0.0212)
Household size	0.00308* (0.00178)	− 0.00393 (0.00366)	− 0.00427 (0.00289)	− 0.0115*** (0.00377)
Share of children in household	0.0153 (0.0171)	0.0488* (0.0248)	0.0470 (0.0300)	0.0559 (0.0384)
Share of elderly (age > 60) in household	0.0467* (0.0263)	− 0.0113 (0.00797)	0.0224 (0.0567)	− 0.0371 (0.0646)
Short-term social assistance (COVID related)	− 0.0117 (0.0264)	− 0.00129 (0.00143)	0.0420 (0.0387)	0.00106 (0.0428)
Health insurance (household)	0.00109 (0.00974)	− 0.0249 (0.0190)	− 0.0298* (0.0158)	− 0.0325* (0.0192)
Observations	3,416	3,416	3,416	3,416
R-squared	0.022	0.029	0.017	0.019

County dummies are included. Standard errors (in parenthesis) are clustered at the county level, * *p* < 0.10, *** p* < 0.05, *** *p* < 0.01

**Table 11:** Robustness check of social assistance on attributes of social cohesion

	Inclusive identity	Cooperation (horizontal)	Trust government	Trust parliament
Social assistance*After first wave of pandemic	0.0493 (0.0383)	0.00790 (0.00965)	0.0472 (0.0459)	0.0592 (0.0589)
After first wave of pandemic	− 0.0167 (0.0194)	− 0.0220** (0.0106)	− 0.0350** (0.0171)	− 0.0169 (0.0144)
Social assistance	− 0.0218 (0.0339)	0.00872 (0.00971)	0.0419 (0.0336)	0.0445 (0.0411)
Age 30–39	0.000404 (0.0264)	0.00272 (0.00337)	− 0.0507** (0.0218)	− 0.0581** (0.0266)
Age 40–49	0.0261 (0.0275)	0.00463 (0.00556)	− 0.00583 (0.0269)	− 0.0648* (0.0331)
Age 50–59	− 0.0311 (0.0320)	0.00455 (0.00653)	− 0.0268 (0.0440)	− 0.0413 (0.0555)
Age > 60	0.0442 (0.0307)	0.0110 (0.00688)	0.00950 (0.0545)	− 0.00818 (0.0730)
Female	0.0431** (0.0170)	− 0.00963 (0.0273)	− 0.0121 (0.0318)	0.0420 (0.0304)
Primary education	0.0846** (0.0344)	0.0191 (0.0122)	0.0360 (0.0595)	0.0591 (0.0658)
Secondary education	0.0336 (0.0368)	0.0170 (0.0128)	− 0.0669 (0.0645)	− 0.0278 (0.0713)
University education	0.0466 (0.0602)	0.00804 (0.0125)	0.0772 (0.0728)	0.0604 (0.103)
Married	0.00751 (0.0172)	− 0.00496 (0.00344)	0.00710 (0.0268)	− 0.00958 (0.0343)
Disability in the household	− 0.0499 (0.0356)	− 0.00514 (0.00456)	− 0.00116 (0.0465)	− 0.0473 (0.0596)
Chronic illness in the household	0.0254 (0.0238)	− 0.00752* (0.00426)	− 0.0373 (0.0255)	− 0.0419 (0.0328)
Household size	0.000891 (0.00354)	− 0.00170 (0.00114)	− 0.0134** (0.00558)	− 0.0186** (0.00745)
Share of children in household	− 0.0426 (0.0352)	0.00274 (0.00712)	0.0670 (0.0573)	0.0856 (0.0690)
Share of elderly (age > 60) in household	0.00848 (0.0364)	− 0.00900 (0.0138)	− 0.0393 (0.0885)	− 0.00342 (0.110)
Short-term social assistance (COVID related)	− 0.00843 (0.0264)	− 0.000718 (0.00144)	0.0396 (0.0387)	− 6.08e-06 (0.0454)
Health insurance (household)	0.000564 (0.0156)	0.000184 (0.00384)	− 0.00277 (0.0280)	− 0.0330 (0.0446)
Wave 2*Age 30_39	− 0.0302 (0.0288)	− 0.00397 (0.00358)	0.0102 (0.0270)	0.0199 (0.0330)
Wave 2*Age 40_49	− 0.0307 (0.0358)	− 0.00597 (0.00588)	0.0140 (0.0291)	0.0330 (0.0427)
Wave 2*Age 50_59	0.0314 (0.0309)	− 0.00563 (0.00728)	0.0270 (0.0527)	0.0220 (0.0667)
Wave 2*Age > 60	− 0.0517 (0.0385)	− 0.00641 (0.00598)	0.00266 (0.0599)	0.0524 (0.0931)
Wave 2*Female	− 0.00400	− 0.000102	0.00629	− 0.0308
	(0.0205)	(0.00306)	(0.0363)	(0.0391)
Wave 2*Primary education	− 0.0572 (0.0383)	− 0.0220 (0.0144)	− 0.0189 (0.0567)	− 0.0595 (0.0671)
Wave 2*Secondary education	− 0.0151 (0.0414)	− 0.0205 (0.0157)	0.0702 (0.0643)	− 0.0166 (0.0751)
Wave 2*University education	− 0.139* (0.0696)	− 0.0127 (0.0149)	− 0.174* (0.100)	− 0.148 (0.116)
Wave 2*Married	− 0.0106 (0.0213)	0.00461 (0.00398)	0.0211 (0.0305)	0.0613 (0.0393)
Wave 2*Disability in the household	0.0243 (0.0436)	0.00908 (0.00546)	− 0.00706 (0.0634)	− 0.00306 (0.0782)
Wave 2*Chronic illness in the household	− 0.0673** (0.0273)	0.00792* (0.00415)	− 0.00907 (0.0365)	− 0.0246 (0.0457)
Wave 2*Household_size	0.00320 (0.00410)	0.00217* (0.00122)	0.0128* (0.00726)	0.00908 (0.00941)
Wave 2*Share of children in household	0.0834* (0.0441)	0.00432 (0.00871)	− 0.0234 (0.0827)	− 0.0519 (0.0881)
Wave 2*Share of elderly (Age < 60)	0.0535 (0.0467)	− 0.00208 (0.0125)	0.0895 (0.126)	− 0.0455 (0.140)
Wave 2*Health insurance (household)	0.000422 (0.0227)	− 0.00380 (0.00356)	− 0.0369 (0.0318)	0.00104 (0.0525)
Observations	3416	3416	3416	3416
R-squared	0.027	0.202	0.022	0.020

County dummies are included. Standard errors (in parenthesis) are clustered at the county level, **p* < 0.10, ***p* < 0.05, ****p* < 0.01

**Table 12:** Robustness check of social assistance effects including county-specific time trends

	(1)	(2)	(3)	(4)
	Inclusive identity	Cooperation (horizontal)	Trust government	Trust parliament
Social assistance*After first wave of pandemic	0.0428 (0.0325)	0.00603 (0.00595)	0.0404 (0.0472)	0.0541 (0.0609)
After first wave of pandemic	− 0.0132 (0.0772)	− 0.0225*** (0.00151)	− 0.0287** (0.0123)	− 0.012 (0.013)
Social assistance	− 0.0229 (0.0555)	0.0108 (0.0115)	0.0452 (0.0783)	0.0480 (0.0410)
Age 30–39	− 0.0195 (0.0124)	2.78e-05 (0.00133)	− 0.0444** (0.0182)	− 0.0436** (0.0214)
Age 40–49	0.00445 (0.0126)	9.95e-05 (0.00164)	0.00303 (0.0189)	− 0.0355 (0.0234)
Age 50–59	− 0.00640 (0.0158)	0.000101 (0.00191)	− 0.00657 (0.0235)	− 0.0181 (0.0284)
Age > 60	0.0107 (0.0201)	0.00641** (0.00282)	0.00791 (0.0327)	0.0301 (0.0388)
Female	0.0403*** (0.0144)	− 0.00113 (0.00178)	− 0.0160 (0.0202)	0.0148 (0.0240)
Primary education	0.0469*** (0.0169)	0.00336* (0.00196)	0.0274 (0.0252)	0.0105 (0.0307)
Secondary education	0.0248 (0.0192)	0.00252 (0.00220)	− 0.0145 (0.0285)	− 0.0474 (0.0344)
University education	− 0.0519 (0.0391)	− 0.000528 (0.00281)	− 0.0406 (0.0470)	− 0.0513 (0.0556)
Married	− 0.00255 (0.0101)	− 0.00189 (0.00119)	0.0234 (0.0155)	0.0332* (0.0185)
Disability in household	− 0.000432 (0.0146)	4.98e-05 (0.00178)	0.00942 (0.0202)	0.000283 (0.0241)
Chronic illness in household	− 0.0326* (0.0189)	0.000803 (0.00192)	− 0.00445 (0.0249)	− 0.0423 (0.0317)
Household size	− 0.0153 (0.0146)	− 0.00205 (0.00158)	− 0.0428** (0.0214)	− 0.0566** (0.0253)
Share of children in household	0.00241 (0.00257)	− 0.000121 (0.000310)	− 0.00611 (0.00402)	− 0.0154*** (0.00475)
Share of elderly (age > 60) in household	0.0216 (0.0222)	0.00597** (0.00265)	0.0589* (0.0350)	0.0704* (0.0415)
Short-term social assistance (COVID related)	0.0425 (0.0315)	− 0.0108** (0.00477)	0.0328 (0.0497)	− 0.0211 (0.0610)
Health insurance (household)	− 0.00739 (0.0200)	− 0.00127 (0.00172)	0.0382 (0.0305)	− 0.00453 (0.0387)
Observations	3416	3416	3416	3416
R-squared	0.058	0.056	0.068	0.074

Standard errors (in parenthesis) are clustered at the county level, **p* < 0.10, ***p* < 0.05, ****p* < 0.01. County dummies and the interaction terms between the county dummy variables and the time indicator have been included in order to consider county-specific time trends

**Table 13:** Balancing test on covariates at baseline (before the pandemic) after applying PSM-kernel matching

Weighted variables	No social assistance (weighted mean)	Social assistance (weighted mean)	Difference	*t* value	Pr(|*T*| >|*t*|)
Age 30–39	0.234	0.228	− 0.005	0.110	0.912
Age 40–49	0.210	0.214	0.004	0.080	0.938
Age 50–49	0.130	0.123	− 0.007	0.180	0.860
Age > 60	0.049	0.047	− 0.002	0.100	0.917
Female	0.462	0.493	0.031	0.520	0.604
Primary education	0.600	0.609	0.009	0.330	0.740
Secondary education	0.241	0.231	− 0.010	0.210	0.831
University education	0.012	0.009	− 0.002	0.250	0.804
Married	0.647	0.669	0.022	0.380	0.702
Disability in the household	0.109	0.115	0.006	0.160	0.876
Chronic illness in the household	0.244	0.214	− 0.030	0.570	0.572
Household size	4.307	4.332	0.025	0.090	0.930
Share of children (age < 15) in household	0.326	0.333	0.007	0.230	0.821
Share of elderly (age > 60) in household	0.075	0.064	− 0.011	0.430	0.666
Health insurance (household)	0.202	0.191	− 0.011	0.240	0.811
*N*	707	105			

**Table 14:** Balancing test on covariates at follow-up (after the first wave of the pandemic) after applying PSM-kernel matching

Weighted variables	No social assistance (weighted mean)	Social assistance (weighted mean)	Difference	*t* value	Pr(|*T*| >|*t*|)
Age 30–39	0.226	0.214	− 0.011	0.33	0.7412
Age 40–49	0.246	0.256	0.01	0.28	0.7827
Age 50–49	0.141	0.139	− 0.002	0.08	0.9337
Age > 60	0.045	0.062	0.017	0.54	0.5908
Female	0.446	0.450	0.014	0.35	0.7258
Primary education	0.605	0.599	− 0.006	0.14	0.8862
Secondary education	0.248	0.240	− 0.007	0.21	0.8347
University education	0.019	0.016	− 0.003	0.30	0.7635
Married	0.629	0.627	− 0.002	0.05	0.9594
Disability in the household	0.097	0.095	− 0.003	0.11	0.9100
Chronic illness in the household	0.188	0.192	0.005	0.14	0.8853
Household size	4.377	4.362	− 0.015	0.08	0.9326
Share of children (age < 15) in household	0.293	0.291	− 0.002	0.07	0.9406
Share of elderly (age > 60) in household	0.102	0.105	0.012	0.53	0.5941
Health insurance (household)	0.244	0.242	− 0.002	0.06	0.9489
*N*	1908	272			

**Table 15:** Robustness check of social assistance on attributes of social cohesion: kernel matching, difference-in-differences analytical framework

Outcome variables	Inclusive identity	Cooperation (horizontal)	Trust in government	Trust in parliament
(${\beta }_{1})$ Social assistance*After first wave of pandemic	0.0480 (0.031)	0.0100 (0.011)	0.0516 (0.0590)	0.0521 (0.0574)
(${\beta }_{2})$ After first wave of pandemic	− 0.016 (0.011)	− 0.0210* (0.0109)	− 0.0401* (0.0231)	− 0.0321* (0.0189)
(${\beta }_{3})$ Social assistance	0.047 (0.045)	0.011 (0.072)	0.0320 (0.0451)	0.0431 (0.0592)
*N*	2992	2992	2992	2992
Adjusted R-squared	0.01	0.03	0.02	0.01

Matching was done on the individual/household variables that were used in specification (1) of the estimation model. County fixed effects are included as well. Standard errors (in parentheses) are clustered at the county level, *, ** and *** denote *p* < 0.10, *p* < 0.05 and *p* < 0.01, respectively

## Sampling Design and Process

The sample universe associated with our survey includes all households in Kenya that operate in the informal economy on the day of the survey. We exclude households that are operating in the formal economy. To obtain a nationally representative cross section of this target population, we use the most recent national census data from the National Bureau of Statistics (KNBS) in Kenya. We used a clustered, stratified, multi-stage, probability sample design. The objective of our sample design was to give every household that operate in the informal economy an equal chance of being chosen for inclusion in the sample. This ensures that the survey provides a representative estimate of the views of the target population. We reached this objective by (a) strictly applying random selection methods at every stage of sampling and by (b) applying sampling with probability proportionate to adult population size.

The sampling process was based on stratification of the country into regions. Regions were further classified into counties and these were further divided into districts and villages. Primary sampling units (PSUs)—sometimes referred to as enumeration areas—are the smallest geographical unit/cluster for which reliable population data were obtainable. The primary sampling units were selected from each stratum based on shares of the national population and number of households, and further allocated based on the urban/rural divide.

The sampling process was structured in four stages and follows largely the process of the Afrobarometer surveys (Afrobarometer Survey Manual 2017): (i) selection of enumeration areas; (ii) selection of sampling start points; (iii) selection of households; and (iv) identifying households that operate in the informal economy for interview.

(i) Selecting enumeration areas (EA): Based on the latest and updated population census Kenyan National Bureau of Statistics (KNBS) randomly select enumeration areas for each stratum and respective rural/urban divide, based on probability proportional to size of population and number of households.

(ii) Selecting the sampling start points (SSPs) for each enumeration area: As no complete lists of households of the informal economy were available from which the sample could be randomly drawn for each EA, we use physical maps of the enumeration areas that were provided by the KNBS. A random sampling start point (SSP) is marked on the map and field teams travel as close as possible to it, or to housing settlements nearest to it. A second SSP is selected as a reserve or substitute in case the initial SSP is inappropriate or inaccessible. Random selection of a start point uses a grid. A ruler is placed along the top of the map and another along the side. A table of random numbers is then used to select pairs of numbers, one for the top axis and one for the side axis, resulting in a random combination. A line is then drawn on the map horizontal to the number chosen on the side, and another line is drawn vertical to the number chosen on the top. The point on the map where these two lines intersect is the sampling start point. Each *x*–*Y* pair of numbers from the random number table can be used only once.

(iii) Selecting the household—walking pattern of interview teams: The interviewers start walking away from the physical startpoint, with interviewer 1 walking towards the sun; interviewer 2 in the opposite direction; interviewers 3 and 4 at a 90-degree angle to the right and left. With this walking pattern, all four directions are covered. By counting households on both sides of the walking path, household No. 5 is selected as the first household for the interview and household No. 15 for the second interview. Special rules were applied in the case of multi-storey buildings, widely scattered households and settlements within commercial farms.

If the interview cannot take place because nobody is at home, or the interview starts but cannot be finished, the walk continues to the next household on the same side of the road or opposite (household No. 6), while the second interview is done in household No. 16.

If the interview is refused the walk continues in the same direction until household No. 15. The second interview would take place with household No. 25.

(iv) Identifying households for the interview that operate in the informal economy: At the household level, each interview is done in two phases. Phase 1 of the interview is conducted with the household head living in the household. The household head provides demographic and employment information on each member of the household (15 or older).

Based on this screening a list is drawn up to include all household members who operate in the formal and informal economy. The interview was ended if at least one member (15 or older) is active in the formal economy and the household was replaced by another household.

For households were no member is active in the formal economy, the respondent for the main part of the interview (phase 2) is randomly selected from the list of persons that operate in the informal economy for interview. If the randomly selected respondent is unavailable the fieldworker makes an appointment for a later time in the day for a second attempt. If the interview is unsuccessful after the second attempt, the fieldworker randomly selects another respondent who qualifies within the same household for the interview. If the second respondent is unavailable or the interview is unsuccessful for whatever reason, the household is dropped and the fieldworker replaces it with another household.

To identify activities within the informal economy, the survey used the following operational definitions: (i) Informal farming, raising animals or fishing: economic activities whose products have been produced for sale were grouped as informal. (ii) Informal employees: paid job with no reference to an employer’s tax contribution or contribution to a public or private pension scheme. If employers did not pay contributions, employees were grouped as informal. (iii) Informal employers and own-account workers: informality is defined by non-registration in the national registry, which is used for company taxation. (iv) Contributing family workers: defined, by default, as having an informal job because of the informal nature of jobs held by contributing family workers that also can include unemployed or students.

## Footnotes

1 The COVID-19 pandemic is a specific type of covariate shock as it affects all members of a society at the same time and can change social cohesion through changes in societal relationships between individuals and the relationship between state and citizens. In contrast, idiosyncratic shocks, such as natural disasters, have devastating impacts on some members of a society, but cannot affect relationships within societies as a whole. Thus, it is of particular relevance to study the mediating effects of social protection on social cohesion during the COVID-19 pandemic.

2 Additional technical support, including data management, was provided by the Institute for Development Studies (IDS), University of Nairobi.

3 The informal economy is defined as all economic activities by economic units that are—in law or in practicenot covered or insufficiently covered by formal arrangements (ILO 2002).

4 The social assistance programmes were not re-targeted due to the pandemic nor were new beneficiaries added to either programme (Doyle and Ikutwa 2021).

5 Coverage of social security programmes, such as social insurances, is limited. Only 3% of informal workers are covered (KNBS 2019). In terms of health insurance, 7.7 million members are covered, but most members are from the formal sector where membership is compulsory (Government of Kenya 2017).

6 On 18 November 2021, the exchange rate for the Kenyan shilling was KES 1 = USD 0.0089 (Onvista 2021).

7 The targeting criteria of the NSNP and the HSNP have not changed during the COVID-19 pandemic.

8 The random selection of the household member was done after screening all household members with the tablet computers that were used during the survey.

9 Random sampling with probability proportional to population size was applied at each stage. The sampling process was based on stratification of the country into regions. Regions were further classified into counties, and these were further divided into districts and villages. Primary sampling units (PSUs) are the smallest geographical unit for which reliable population data are obtainable. The primary sampling units were selected from each stratum based on its share of the national population, and further allocated based on the urban/rural divide. Twice as many primary sampling units were selected from lockdown counties to enable a detailed analysis. This oversampling was accounted for by applying sampling weights in the subsequent analysis.

10 NSNP and the HSNP have been continued during the pandemic and their targeting criteria have not been changed.

11 Respondents are household members over the age of 15. They were randomly selected from the household after the screening of all household members. The random selection was done with the tablet computers that were used during the survey. As the analysis relies on cross-sectional surveys, respondents were not surveyed twice.

12 Descriptive statistics of all explanatory variables can be found in Table 6 of the Appendix. Tables 7 and 8 in Appendix shows the means of all explanatory variables by social assistance coverage before and after the pandemic.

13 Please note that these robustness checks cannot replace the missing analysis of the parallel trend assumption.

14 Given the set of individual and household characteristics, the estimation results are based on 3,416 of 3,796 household heads on whom we have complete information on all variables.

15 *F*-tests were conducted in order to explore whether the estimated effects in lockdown and non-lockdown counties for social assistance beneficiaries and non-social beneficiaries are jointly statistically different (test for the joint significance of shift in intercept and slope). The tests show a joint significance of the estimated effects in lockdown and non-lockdown counties for the trust in government and cooperation outcome variables at the 10% level.

**Table 5:** Heterogeneity of social assistance effects due to differences in regional lockdown policies

Outcome variables	Trust parliament	Trust government	Inclusive identity	Cooperation (horizontal)
(${\beta }_{1})$ Social assist*After pandemic*Lockdown	0.02 (0.09)	0.03** (0.01)	0.01 (0.07)	0.04** (0.02)
${(\beta }_{2})$ After pandemic * Lockdown	− 0.10*** (0.03)	− 0.05* (0.03)	− 0.05*** (0.01)	− 0.02 (0.02)
${(\beta }_{3})$ After pandemic*Social assistance	0.01 (0.07)	0.02 (0.06)	0.03 (0.05)	0.01 (0.01)
*N*	3416	3416	3416	3416
adj. R-sq	0.01	0.01	0.06	0.03

Control variables and county dummies are included. Standard errors (in parenthesis) are clustered at the county level. **p* < 0.10, ***p* < 0.05, ****p* < 0.01

16 In general, the increase in cooperation with others was possible in lockdown counties, as the containment measures did not include stay-at-home restrictions and the survey was conducted six months after the ease of lockdowns in these counties. So these activities could also have been done after the lockdown policies have been ended.

17 Relief measures and adapted social assistance were frequently covered in public speeches and press statements by the government broadcasted over radio and television during the pandemic (Government of Kenya 2020; Ministry of Health – Kenya 2020), so it is likely that the beneficiaries perceive the state as the main implementer of these programmes.
